# The impact of COVID‐19 on work, training and well‐being experiences of nursing associates in England: A cross‐sectional survey

**DOI:** 10.1002/nop2.928

**Published:** 2021-05-10

**Authors:** Rachel King, Tony Ryan, Michaela Senek, Emily Wood, Bethany Taylor, Angela Tod, Ashfaque Talpur, Steve Robertson

**Affiliations:** ^1^ Division of Nursing and Midwifery Health Sciences School The University of Sheffield Sheffield UK

**Keywords:** COVID‐19, cross‐sectional survey, trainee nursing associates, training, well‐being, workforce

## Abstract

**Aim:**

To explore how the COVID‐19 pandemic affected nursing associate work, training and well‐being experiences.

**Design:**

Cross‐sectional survey.

**Methods:**

A survey of trainee and newly qualified nursing associates was completed in July 2020. Closed responses were analysed using descriptive statistics with inferential comparisons made between community and secondary care settings. Open questions were analysed thematically.

**Results:**

Sixty‐four participants responded. Over half (53.2%) experienced an increased workload with 24.2% reporting extensions in their role. One third (32.3%) were redeployed, and a quarter (24.2%) did not feel safety concerns were adequately addressed when raised. Those working in the community reported significantly more concerns about staffing (*p* = .03), working overtime (*p* = .03), missed care (*p* = .02) and safety (*p* = .04). Despite this, many (75.8%) participants felt able to provide the same standards of care. Several spoke about enhanced teamwork, and the majority (96.8%) were not looking to leave their post.

## 1 INTRODUCTION

The nursing associate (NA) role was developed in England as a bridge between healthcare assistants and Registered Nurses and as a strategy to address the nursing workforce shortage (Health Education England, 2015), described as one of the greatest threats to the health service (Kings Fund, 2018).

Severe acute respiratory syndrome coronavirus 2 (SARS‐CoV‐2), known as COVID‐19, has undoubtedly increased this threat, exacerbating the demand‐capacity gap in staffing levels. Healthcare workers are at greater risk of catching the virus, particularly in areas where personal protective equipment (PPE) is inadequate (Bielicki et al., 2020).

As yet, little work has explored how COVID‐19 is affecting nursing associates. This paper focusses on the impact that the pandemic is having on this new occupational group's experiences, in order to enhance support and development in ways that promote recruitment and aid retention.

## 2 BACKGROUND

The first nursing associates (NAs) were introduced to the healthcare workforce in England in 2019 with the aim of bridging the gap between unregistered healthcare assistants and registered nurses and to provide an alternative route into nursing (Health Education England, 2015). NAs undertake a 2 year training programme, prior to registering with the Nursing and Midwifery Council, and are required to adhere to national standards of proficiency (Nursing & Midwifery Council, 2018). Similar roles have been deployed in countries with comparable healthcare systems such as the US, Canada, Australia and New Zealand (The Health Foundation, 2016). However, as yet, little research has been conducted around this new role in England.

Since the identification of a new coronavirus in December 2019, the number of cases has rapidly increased and spread across the globe (Li et al., 2020). COVID‐19 is placing a significant burden on healthcare services worldwide, with concerns amongst healthcare professionals about increased workload, adjusted working practices, and risk of exposure to themselves and their families, leading to workforce shortages and burnout (Azzopardi‐Muscat, 2020; Heath et al., 2020).

Healthcare workers caring for patients with COVID‐19 are at higher risk of contracting the virus than the general population (Salas‐Vallina et al., 2020; Shah et al., 2020), with Black, Asian and minority ethnic (BAME) individuals at particularly high risk (Moorthy & Sankar, 2020; Salisbury, 2020). A study by Shah et al. (2020) in Scotland found that the risk of hospital admission due to COVID‐19 was threefold for healthcare workers and twofold for members of their households. Furthermore, 17.2% of all admissions due to COVID‐19 were healthcare workers or their households (Shah et al., 2020).

Concerns amongst healthcare workers are exacerbated by shortages of personal protective equipment (PPE) (Bielicki et al., 2020; Newman, 2020) and psychological distress (Heath et al., 2020; Moorthy & Sankar, 2020). Studies of previous respiratory pandemics have also noted concerns about PPE and infectivity (Koh et al., 2012; Lam & Hung, 2013). The impact of the pandemic on frontline staff is clear to those in senior management. A survey of 199 hospital executive directors across England revealed that 99% are concerned about staff burnout following the first wave of COVID‐19 (NHS providers, 2020), with fears of an impending “perfect storm” of workforce shortages, staff burnout and the second wave of coronavirus.

A narrative review of similar outbreaks (SARS‐1 and Ebola) by Heath et al., (2020) identified several strategies to reduce the psychological impact on the healthcare workforce. For example, self‐care, a supportive workplace culture, adequate PPE and appropriate training when redeployed to other areas. They concluded that effective leadership, resilience training and psychological first aid could help counter psychological distress for healthcare workers during a pandemic. Similarly, Mills et al. (2020) advise that, in order to respond to a crisis such as a pandemic, workforce well‐being and resilience depend on self‐care and staff support through compassionate leadership. The importance of supportive leadership was revealed in a survey in Hubei China, which found that nurses were more likely to volunteer to care for COVID‐19 patients if they were provided with adequate training and psychological support (Gan et al., 2020). The impact of effective leadership was also highlighted in a study of healthcare leaders in Spain, where rates of COVID‐19 in the workforce appeared lower in settings with a high level of trust and information sharing (Salas‐Vallina et al., 2020).

Improving recruitment and retention has been a UK healthcare workforce priority for some time (NHS Improvement, 2019). Previous studies have identified reasons for student nurse attrition and nurse dissatisfaction. Student nurse attrition is due to lack of support, unmet expectations and financial concerns (Health Education England, 2018). Furthermore, nurses have reported feeling demoralized from a perceived lack of support and staff shortages (Senek et al., 2020). Future key priorities for the workforce are to improve the experience of providing care, including prioritizing mental health and psychosocial support (Azzopardi‐Muscat, 2020). Organizations have also been prompted to consider how best to protect healthcare workers and their families to maintain services and reduce community transmission of COVID‐19 (Moorthy et al., 2020; Shah et al., 2020).

While previous work on the effect of COVID‐19 on the nursing workforce is clearly important, little is yet known about how COVID‐19 has affected nursing associates in relation to their work, training and well‐being. This study focusses on how the pandemic is being experienced by this new occupational group during the first wave of the pandemic.

### 2.1 Research question

How is COVID‐19 affecting the work, training and well‐being experiences of nursing associates?

## 3 THE STUDY

### 3.1 Design

The study used a cross‐sectional survey design with closed and open questions to examine how COVID‐19 is affecting the work, training and well‐being experiences of trainee nursing associates (TNAs).

### 3.2 Methods

A purposive sample of TNAs across England was recruited to a longitudinal cohort study in 2019. Recruitment was facilitated through university email lists; course leaders in seven Higher Education Institutions (HEIs) across England were contacted via email and asked to disseminate information about the study to their TNA student groups. Social media twitter posts were also used to recruit TNAs to this cohort. The HEIs were purposively chosen to provide geographical spread across England and diversity in approach to NA training, thereby helping reduce bias that might come from a limited geographical sample or from a single HEI establishment. Social media recruitment further extended this geographical and training diversity.

In July 2020, a specific survey on COVID‐19 was distributed to this established cohort of 121 TNAs. Informed by issues of concern emerging in the literature on respiratory pandemics, and contemporary concerns reported in the nursing press and highlighted by UK professional nursing organizations (such as the Royal College of Nursing), a draft survey was developed and further refined through research team discussion. The final version of the survey included questions on participant characteristics and 26 open (free text) and closed (forced choice) questions on the TNA/NAs experiences of COVID‐19 across six domains (See Box 1). The survey was compiled using Google forms and sent to email addresses provided by cohort participants.

BOX 1 **Survey Question Domains**

- Preparedness for dealing with COVID‐19 (PPE, staff numbers, training)
- Feeling safe at work (and if exposure could have been reduced)
- Nature of and ability to raise concerns (and these being actioned)
- Change in tasks and workload (including moving clinical area, change in role, impact on training, working additional overtime)
- Provision of care (standard of care, face‐to‐face contact with COVID‐19 patients, most valuable contribution)
- Intention to leave

## 4 ETHICS

Research Ethics Committee approval was gained from the University of Sheffield Research Ethics Committee (reference 026355). A link to the information sheet was included in the email sent out by the HEIs and in the social media advert, with a further link to the consent form on the information sheet. Informed consent was gained from participants prior to survey completion.

### 4.1 Rigour

A number of strategies were used to ensure rigour. Twice‐weekly meetings were held between the first three authors throughout the study to critically consider issues, and research team meetings were held to facilitate additional reflection and input at key points (e.g. commenting on design, on survey questions and assisting with theme development). Colleagues external to the project but involved with NA training were consulted at various stages to help sense check: recruitment processes, survey questions and emerging findings. Data collection involved data source triangulation (from several HEIs and via social media). Coding, theme development and integration and interpretation of data were checked independently and collaboratively by the research team (see analysis section). We have used the “Strengthening the Reporting of Observational Studies in Epidemiology” (STROBE) reporting guideline for cross‐sectional studies (von‐Elm et al., 2007).

### 4.2 Analysis

Two researchers analysed the quantitative data (TR and MS). Descriptive statistics have been used to present participant demographics. Descriptive and inferential (Chi square) statistics were used to analyse the quantitative measures. For the purpose of this paper, we divided participants into those who worked in (primarily hospital‐based) acute care contexts (*n* = 22, 35%) and those who worked in community contexts (*n* = 40, 65%). Two participants did not provide sufficient information to locate them in either of these contexts and have therefore been removed for the quantitative analysis (though their responses remain included in the qualitative analysis). Differences and similarities in the quantitative measures for participants working in the acute and community sector are considered given the potential for different experiences of COVID‐19 in these two settings. SPSS 25 software was used to support this stage of analysis.

Answers to open ended questions have been analysed thematically using the six steps outlined by Braun and Clarke (2006). Initial coding was undertaken independently by the first author (RK) and sections of this checked SR. Further coding, categorizing and initial theme development was completed collaboratively by these three co‐authors and then final themes refined in discussion with the whole research team. Quirkos^©^ computer assisted qualitative data analysis software (CAQDAS) version 2.3.1 was used to manage the qualitative data.

The closed and open question design was used to facilitate the development of both data patterns and in‐depth insights into the experiences of COVID‐19 on this sub‐section of the healthcare workforce. Data were integrated through iterative consideration of both data sets collectively by four members of the research team (RK, SR, TR and MS) in order to generate the critical discussion.

## 5 RESULTS

There were 64 responses to the survey, a response rate of 53%. Participant demographics are presented in Table 1. Most participants were female (*n* = 61%, 95%), white British (*n* = 61%, 95%) with a range of ages, and representation from both newly qualified NAs (*n* = 21%, 33%) and TNAs (*n* = 43%, 67%).

**TABLE 1:** Demographics of participants (*n* = 64)

Category	Group	Number (*n*) Percentage^a^ (%)
Gender	Female	61 (95%)
	Male	3 (5%)
Age	21–30	21 (33%)
	31–40	25 (39%)
	41–50	12 (19%)
	51–60	5 (8%)
Ethnic background	White British	61 (95%)
	White Other	1 (2%)
	Black African	2 (3%)
Occupation	Trainee (TNA)	43 (67%)
	Nursing Associate (NA)	21 (33%)
Setting	Acute Care	22 (35%)^b^
	Community Care	40 (65%)^b^

^a^ These % have been rounded to the nearest whole number.

^b^ Two participants did not provide sufficient information to locate them.

Participants worked across England in a wide range of healthcare services including adult, children's, mental health, learning disabilities, community and primary care, with the majority based in adult general and surgical settings.

The responses to the closed questions are presented in Table 2 and described below.

**TABLE 2:** Experiences of TNA/NAs at work during the COVID‐19 pandemic

Variable Outcome	All Number (%)		Community Care Number (%)		Acute Care Number (%)		Significance
	Yes	No	Yes	No	Yes	No	
Did you care for COVID patients face to face? (Y_N)	44 (71)	18 (29)	29 (72.5)	11 (27.5)	15 (68.2)	7 (31.8)	*p* = .7
Preparedness (PPE) Did you have enough PPE? (Y_N)	40 (64.5)	22 (35.5)	25 (62.5)	15 (37.5)	15 (68.2)	7 (31.8)	*p* = .4
Preparedness (Staff Training) Did staff get enough training? (Y_N)	33 (53.2)	29 (46.8)	19 (47.5)	21 (52.5)	14 (63.6)	8 (36.4)	*p* = .17
Preparedness (Staff Numbers) Were there enough staff? (Y_N)	34 (54.8)	28 (45.2)	18 (45)	22 (55)	16 (72.7)	6 (27.3)	χ2 (1) = 4.406, *p* = .036*
Overtime Work Did you have to work more overtime than normal? (Y_N)	28 (45.2)	34 (54.8)	22 (55)	18 (45)	6 (27.3)	16 (72.7)	χ2 (1) = 4.406, *p* < .036*
Missed Care/Care Left Undone I do not have enough time to get things done (Y_N)	32 (51.6)	30 (48.4)	25 (62.5)	15 (37.5)	7 (31.8)	15 (68.2)	χ2 (1) = 5.350, *p* = .021*
Has the COVID−19 pandemic affected your job role? (Y_N)	39 (62.9)	23 (37.1)	27 (67.5)	13 (32.5)	12 (54.5)	10 (45.5)	*p* = .3
Have you extended your role beyond usual scope? (Y_N)	15 (24.2)	47 (75.8)	12 (30)	28 (70)	3 (13.6)	19 (86.4)	*p* = .2
Have you been asked to work in a different workplace than normal? (Y_N)	20 (32.3)	42 (67.7)	10 (25)	30 (75)	10 (45.5)	12 (54.5)	*p* = .15
Could you raise concerns with employer if risk to staff or patient? (Y_N)	56 (90.3)	6 (9.7)	36 (90)	4 (10)	20 (90.9)	2 (9.1)	*p* = .9
Were appropriate actions taken when concerns were raised? (Y_N)	47 (75.8)	15 (24.2)	29 (72.5)	11 (27.5)	19 (81.8)	4 (18.2)	*p* = .3
Did you feel safe at work during pandemic? (Y_N)	44 (71)	18 (29)	25 (62.5)	15 (37.5)	19 (86.4)	3 (13.6)	χ2 (1) = 3.923, *p* = .048*
Do you think your COVID Risk could have been reduced? (Y_N)	20 (32.3)	42 (67.7)	16 (40)	24 (60)	4 (18.2)	18 (81.8)	*p* = .07
Were you able to provide the same standard of care for your patients? (Y_N)	47 (75.8)	15 (24.2)	30 (75)	10 (25)	17 (77.3)	5 (22.7)	*p* = .6
Did you want to leave work/training due to COVID? (Y_N)	2 (3.2)	60 (96.8)	2 (5)	38 (95)	0	22 (100)	*p* = .4

A total of 44 (71%) participants said they cared directly for patients with COVID‐19. While 40 (64.5%) participants felt they had sufficient PPE, only 33 (53.2%) felt staff had received enough training. These figures were similar across both acute and community settings. While approximately half of the participants (54.8%) felt adequately prepared in terms of staff numbers, there was a significant difference (*p* = .03) between those in community care (45%) and those in acute care settings (72.7%). It is possible that these challenges about staffing led to 28 (45.2%) participants reporting working more overtime than normal and 32 (51.6%) not having enough time to get work done. Staff in community settings reported working significantly more overtime (55%) than those in acute settings (27.3%) (*p* = .03). Significantly more staff in community settings reported not having sufficient time to complete tasks (62.5%) compared to acute settings (31.8%) (*p* = .02).

Of the participants, 39 (62.9%) stated that COVID‐19 had affected their job role. A question about whether workload had increased, stayed the same or decreased was asked (not presented in the table as it does not have a binary outcome measure). The majority, 43 (69.4%), did notice a change in their workload with 33 (53.2%) experiencing an increase in workload and 9 (14%) a reduction. Fifteen participants (24.2%) had extended their role beyond their normal scope of practice. Additional analysis showed that amongst those who had extended their role only eight were given additional training while five said that training would have been helpful and two that additional training was not required. Twenty (32.3%) had been moved to a different workplace. While 56 (90.3%) felt able to raise concerns with their employer, only 47 (75.8%) felt that appropriate action was taken when they raised concerns. None of these issues around job role showed significant differences between the acute and community sector participants.

While 44 (71%) participants said they felt safe at work during the COVID‐19 period, there was a significant difference between community settings (62.5%) and acute settings (86.4%) (*p* = .04). Linked to this, 20 (32.3%) participants felt that COVID‐19 risk could have been reduced, although this did not quite reach the level of significance between community settings (40%) and acute settings (18.2%) (*p* = .07).

Despite these challenges, 47 (75.8%) participants reported still being able to provide the same standard of care and 60 (96.8%) did not want to leave their training/job. Both of those that stated they did wish to leave worked in a community setting.

### 5.1 Qualitative themes

Three themes were developed from the open questions relating to the impact of COVID‐19 on working practices, training and development, and well‐being experiences.

### 5.2 Impact of COVID‐19 on working practices

Participants responded to the service demands caused by the pandemic. They broadened their scope of practice to care for very unwell patients, worked in new areas through redeployment, drew on experiential knowledge, demonstrated knowledge translation and met the extra demands caused by staff shortages:

The role of the ward I was on changed but I was able to use my knowledge from previous placements to help and assist on the ward which meant stepping up. TNA 4

While some reported these experiences of “stepping up,” others reported the reverse, an enforced move back into healthcare assistant roles:

Staff members being moved and leaving ward staff short so you're fulfilling a health care support worker role. TNA 13

As highlighted earlier, many participants noted changes to their workload. Those that reported an increased workload described a number of reasons for this. Staff shortages (including increased sickness absence), time associated with wearing personal protective equipment (PPE), testing patients for COVID‐19 and caring for a greater number of acutely ill patients requiring more frequent observation and care:

I’m caring for more unwell patients which need further support and there was an increase in workload with associated staff illness which was not always backfilled. TNA 4

Those who experienced a reduction in their workload explained that they cared for fewer patients face to face because of a move to remote consultations and consequently felt their skills were not being used:

Phone assessments were the norm. I can't do those so I spent some days not doing any clinical work. TNA 28

In addition, participants also described variations to their role such as increased cleaning and administrative duties, and meeting the increased support and communication needs of their patients:

I have to clean and disinfect the room, surfaces and equipment after every patient [...] I have to deal with the increasing anxieties of the patients that are worried about coming to clinic. TNA 39

Participants described how they supported worried patients who were unable to receive visitors. Some felt out of their depth in having to care for very unwell patients. Despite this, there was a pride in some of the adjustments they had made to working practices, particularly around being more involved in supporting patients and relatives through such extenuating circumstances. There was a determination to continue providing the best care and maintain usual standards of care:

We had one young man that came for an emergency operation, he was overwhelmed and wanted his family there. Due to Covid this couldn’t happen. I reassured him, told him I would be there throughout his operation. I went to see him on the ward after. TNA 33

I was holding the hand of a frightened patient and comforting him while he sadly passed away due to Covid as his family was unable to be on the ward. TNA 16

However, this ability to maintain standards of care may only have been sustained through high levels of overtime noted earlier, particularly for those working in the community sector:

I went over and above to ensure my patients received the best care available at the time. TNA 19

The TNA/NAs adapted their role to meet the needs of healthcare services during the pandemic, such as substituting for staff shortages through redeployment, taking on cleaning responsibilities to improve infection prevention and control, and providing significant emotional care (as well as physical care) to patients.

### 5.3 Impact of COVID‐19 on training and development

The COVID‐19 pandemic significantly affected participant experiences of training and development. Many trainees faced reduced learning opportunities, cancellation of placements and even the suspension of their training:

Local trust who hold my honorary contracts for placements have suspended placements trust‐wide. TNA 3

Previous to Covid it was already quite a struggle to get clinical skills experience…so I have been reliant on alternative placements to meet the skills required. Unfortunately, one alternative placement was cancelled. Covid has impacted on the limited amount of training that I can do within my base placement e.g BM training [blood glucose monitoring]. TNA 37

Some trainees were unable to develop skills essential to their generic role. For those whose placements continued, staff were often too busy to supervise the development of clinical skills and patient contact was limited:

The registered nurses were too busy to give me learning opportunities so I wasn't able to perform any clinical tasks. TNA 34

Greatly reduced patient contact to zero. TNA 30

University delivery of the academic aspect of training was moved to remote learning which some trainees found difficult:

University has been difficult as I learn best in a face‐to‐face environment with my cohort and peers, Zoom lectures are just not the same. TNA 19

Trainees clearly valued the face‐to‐face weekly university teaching and the associated peer support from other TNAs. They missed this contact with their peers and, to some extent, their lecturers. This coincided with feeling more isolated in their clinical settings, due to infection control procedures and redeployment:

Patients relied on us to be their support, we were alone and so were they. TNA 29

Despite the interruptions to their training and development, some participants spoke positively about their role in supporting other members of their team:

I was able to complete paperwork so that clinicians can go on to do other assessments, and I offer a listening ear to people struggling with lockdown. TNA 28

Furthermore, one participant explained how her generic NA training had equipped her to care for COVID‐19 patients in mental health settings when most of her colleagues lacked relevant skills, as she had recently undertaken training in caring for patients with physical health problems:

I was also able to provide a lot of support and guidance to my RMN colleagues who have had little or no experience of physical health care. Due to my role being generic I was able to utilise experience and skills I had learnt during my training. NA 16

This demonstrates how the generic NA training enabled some participants to provide effective patient care across settings during the pandemic. The impact of COVID‐19 on working practices and on training and development also linked to the effect it had on well‐being for the trainee and newly qualified nursing associates.

### 5.4 Impact of COVID‐19 on well‐being

As noted earlier, the majority of participants stated that they felt safe at work. This, however, masks a number of concerns affecting the well‐being of participants. These include burden of workload, uncertainties attached to being moved to new areas, the disappointment in suspension or reduction of training, the worry of keeping up to date with policy changes, unreliable guidance on and provision of PPE, and associated anxieties around protecting themselves, their families and patients from infection. These all contributed to feelings of stress and fear.

There was widespread confusion early in the pandemic caused by ever‐changing guidance about the correct use of personal protective equipment (PPE):

Initially we were told we only needed to use PPE with confirmed cases. As the virus progressed it became apparent this was not the case, however by then it was too late and we had either been exposed and become ill, or had carried the virus to other patients. NA 3

Furthermore, some felt that colleagues were not adhering to guidelines, and there was a general fear of the unknown. While the majority felt able to raise concerns about this and other risks to staff and patients, many did not feel that appropriate action was taken when concerns were raised leaving them feeling isolated and vulnerable:

I had a very nasty experience within my work team which resulted in me whistleblowing […] I suffered ostracism from my team, confrontation from angry nurses etc. TNA 19

A small number of participants believed they had contracted COVID‐19 while at work. Fear of catching the virus and passing it on to patients or family members was present in many responses, alongside uncertainty about how the disease will play out:

I had serious concerns for my safety as my father has died from Covid and my mother also contracted it and they work in the same Trust as me. NA 8

Such concerns were more apparent for those in the community care sector, those who felt vulnerable due to pregnancy, pre‐existing conditions, or living with clinically vulnerable relatives, and for those who felt there was inadequate provision of PPE:

I have extensive past medical history. Nothing was investigated to see if I was safe to work on a Covid unit. TNA 41

Not having enough PPE has caused me to feel stressed. NA 14

There were some factors that helped provide a counter‐balance to these fears and anxieties and helped foster elements of positive well‐being during these challenging circumstances. The pride in their work noted in the previous section was one such element. Improved teamwork and bonding, feeling that they had an important role to play, were also important positive factors mentioned by several participants:

Although it was difficult we still pulled together as a team, supporting each other and the young people […] I think as a team we are able to acknowledge that the reason we work so well is that everyone has a part to play. TNA 37

Overall, the effect of COVID‐19 on well‐being was therefore mixed, with TNAs/NAs demonstrating a high degree of stress, fear and anxiety while also reporting opportunities for increased pride and sense of connection with colleagues and patients.

## 6 DISCUSSION

Integrating closed and open questions provided a depth of insight into the experiences of the participants. In exploring how COVID‐19 influences the work, training and well‐being of nursing associates, this study highlights several important issues. With the few exceptions noted that showed statistical significance, it is clear that these issues were largely present for TNAs/NAs across both acute and community contexts. These issues, and differences across these two contexts, are discussed below.

First, staff shortages, time‐consuming infection control procedures and caring for patients with higher acuity have increased the workload for many. This resulted in some extending their scope of practice, working more overtime than normal and yet still reporting insufficient time to get necessary work completed. These latter two were particularly noticeable in the community sector where the withdrawal of face‐to‐face care by other services and hurried hospital discharges have added to community nursing pressures during the COVID‐19 crisis (Green et al., 2020). For those whose workload reduced during the pandemic, there was frustration that their skills were not being fully used in supporting care. This has significance as helping colleagues is recognized as a motivator for commencing NA training (Coghill, 2018; King et al., 2020). Future research could consider developing redeployment models that aim to maximize skill utilization.

Second, previous work has shown that during the development and implementation of this new, second level, nursing associate qualification, students and staff often feel in a liminal space with role and task boundaries often being unclear (Davey, 2019; King et al., 2020). Findings here suggest that COVID‐19 accentuated this. Some enjoyed extending the scope of their practice, taking on and learning new skills, others felt unsafe and underprepared in terms of training and equipment as their work or care setting changed. It is possible that extending the scope of practice for this group of trainees was a pragmatic response. However, future research should explore not only the adequacy of support in such circumstances, but also if such extensions of practice breach regulations and the safe standards required during training and postregistration.

Third, COVID‐19 affected participants’ training and development. Decreases in patient contact, discontinuation of clinical placements, reduced supervision and redeployment were all reported. TNAs already face challenges in their training relating to clinical support and career development opportunities (King et al., 2020) and in relation to “worker” versus “learner” perceptions (Coghill, 2018). These have been compounded by COVID‐19. The training of new NAs is seen to be crucial in meeting future healthcare service demands (Health Education England, 2015); therefore, a pause in training as a result of the pandemic has implications for this policy. Furthermore, participants described feeling isolated from their peers as a result of remote university learning. This has relevance as previous work highlights the significance of peer support to trainee NAs as they seek to develop a shared occupational identity (Coghill, 2018; King et al., 2020).

Fourth, the COVID‐19 situation took its toll on the well‐being of this group. Fear about catching and transmitting the virus to family members, noted in previous viral, respiratory pandemics (Koh et al., 2012; Lam & Hung, 2013), were compounded by the stress and anxiety of inadequate PPE provision and, for some, by a perceived lack of action being taken when safety issues were raised. Some of these safety fears were greater in the community sector compared to acute care settings. As others have noted (Green et al., 2020), in the early stages of the pandemic the discharge of patients, with often unknown COVID‐19 status, increased workload and risk anxieties for those working in this sector. Our findings support this, with insufficient staffing and higher levels of missed care (despite higher levels of overtime) being present for community sector participants. These are important issues given that missed care and failure to have safety concerns actioned are two of the main determinants of job dissatisfaction for nurses with a potential impact on intention to leave (Senek et al., 2020). Further strategies to improve the well‐being of this workforce in the context of COVID‐19 could include prioritizing mental health support (Azzopardi‐Muscat, 2020).

Finally, the majority of participants felt able to retain the same standards of care. Many felt proud of the contribution they were making and reported enhanced feelings of cohesion and teamwork. Salas‐Vallina et al., (2020) found that services with greater social capital experienced a lower rate of COVID‐19 infection when compared to those with lower levels of trust and information sharing, highlighting the importance of teamwork and strong enabling leadership.

These findings have implications, particularly for nurse managers and those within the HEI sector with responsibility for TNAs and NAs. Understanding their experiences of TNAs and NAs is vital to both recognizing their support needs and in helping ensure these needs are met. Nurse managers, and those in HEI settings, can be proactive in ensuring safe and appropriate skill utilization. They are well‐placed to provide clear information and help alleviate perceived and real concerns about safety in a rapidly changing situation. They are also in a strong position to activate mental health support, reduce feelings of isolation and facilitate group camaraderie and peer support, all of which help promote resilience for this part of the workforce in this challenging context.

### 6.1 Limitations

The majority of TNAs/NAs who completed this survey were female and white British. Future recruitment to TNA/NA workforce studies should aim to increase the diversity of participants. A further limitation was the response rate of 53%. This can be partly explained by the fact that, despite a request for more permanent contact details, on joining the cohort study in 2019, some participants only provided university email addresses some of which were no longer in use for the 2020 period of data collection. Finally, this limited response rate means the inferential analysis was most likely underpowered. However, we believe these results still give indication as to where future policy and research might focus.

## 7 CONCLUSION

As a new role in the healthcare workforce, nursing associates already face significant challenges which have been exacerbated by COVID‐19. Despite changes in working practices, interrupted training and development, and fear of exposure to COVID‐19, participants strived to provide excellent care and support for their patients and colleagues, sometimes at personal cost. Future strategies to assist NAs as COVID‐19 continues should focus on protecting their development, utilizing their skills in appropriate ways, ensuring adequate support for psychological well‐being and safety and emphasizing their value in the wider healthcare team.

## CONFLICT OF INTEREST

The authors have no conflicts of interest to declare.

## AUTHOR CONTRIBUTIONS

All authors have met the following criteria for authorship:

Have made substantial contributions to conception and design, or acquisition of data, or analysis and interpretation of data;Been involved in drafting the manuscript or revising it critically for important intellectual content;Given final approval of the version to be published. Each author should have participated sufficiently in the work to take public responsibility for appropriate portions of the content; andAgreed to be accountable for all aspects of the work in ensuring that questions related to the accuracy or integrity of any part of the work are appropriately investigated and resolved.

## ETHICAL STATEMENT

Research Ethics Committee approval was gained from the University of Sheffield Research Ethics Committee (reference 026355). A link to the information sheet was included in the email sent out by the HEIs and in the social media advert, with a further link to the consent form on the information sheet. Informed consent was gained from participants prior to survey completion.

## Data Availability

The data that support the findings of this study are available from the corresponding author upon reasonable request.
